# Improving the Accessibility of Consumer Orientation: Co‐Designing Infection Control Training for Consumers Partnering With Health Services

**DOI:** 10.1111/hex.70792

**Published:** 2026-07-28

**Authors:** Clare Soeters, Emmah Doig, Hannah Olufson, Kimberley A. Baxter, Ruth King, Olivia Sarri, Joanne Sherring, Lisa Anemaat

**Affiliations:** ^1^ Faculty of Health, Medicine and Behavioural Sciences, School of Health and Rehabilitation Sciences The University of Queensland Brisbane Queensland Australia; ^2^ Surgical Treatment and Rehabilitation Service (STARS) Education and Research Alliance The University of Queensland and Metro North Health Brisbane Queensland Australia; ^3^ Dietetics & Food Services, STARS, Metro North Health, Queensland Health Brisbane Queensland Australia; ^4^ Surgical Treatment and Rehabilitation Service (STARS), Metro North Health Brisbane Queensland Australia; ^5^ Queensland Aphasia Research Centre The University of Queensland Brisbane Queensland Australia

**Keywords:** CCI/PPI, codesign/coproduction, lived experience, mandatory training, qualitative

## Abstract

**Background:**

Consumer and community involvement (CCI) can enhance research and healthcare outcomes. However, orientation processes, including mandatory training, can pose barriers when not designed for consumers, particularly those with diverse communication, language, and literacy needs.

**Objectives:**

To (1) explore consumer and staff experiences of hospital‐based orientation, including existing infection control training materials; and (2) co‐design an accessible infection control mandatory training resource for consumers who partner with the hospital.

**Design:**

Qualitative study using semi‐structured interviews (consumers and staff) and a series of three co‐design workshops (consumers, staff, researchers).

**Setting and Participants:**

The study was conducted at a metropolitan rehabilitation hospital in Australia in 2025. Participants included consumers, staff and researchers with knowledge in consumer partnering, infection control and orientation.

**Variables Studied:**

Experiences of consumer orientation, including infection‐control training and priorities for the design, content, accessibility and acceptability of a co‐designed infection‐control training for consumers.

**Outcomes Measured:**

Qualitative themes relating to barriers and enablers in consumer orientation and characteristics of the co‐designed training resource.

**Results:**

Nine participants completed an interview, and 14 contributed to co‐design workshops. Consumers described orientation as inflexible, insufficiently tailored and sometimes inaccessible, particularly for people with diverse needs. They valued orientation as an opportunity to build relationships, ask questions and understand their role. Staff highlighted that processes lacked the flexibility needed for person‐centred partnering and described challenges in tailoring information for consumer audiences. Interview findings informed co‐design workshops in which consumers, staff and researchers developed and refined an infection control training resource for consumers in a hospital setting. Key design features included plain language, visual supports, concise content, opportunities for questions and accessible formatting.

**Discussion:**

Consumer orientation is an important but underexamined stage of healthcare partnering. Accessible, flexible and consumer‐centred processes may support inclusive and meaningful involvement. Contributions from staff and consumers were valuable in ensuring that the end product was fit for purpose from organisational and end‐user perspectives.

**Conclusion:**

Orientation and mandatory training may create barriers when not tailored to consumers' needs. Co‐designing accessible training resources offers a practical approach to improving consumer orientation and understanding of infection control principles, supporting more inclusive partnerships with health services.

**Lived Experience or Public Contribution:**

This study was initiated in response to feedback from a consumer network at a metropolitan hospital in Queensland, Australia. Consumers contributed as interview participants and co‐designers in developing the training resource.

**Abbreviations**
CALD: culturally and linguistically diverse
CCI: consumer and community involvement
COREQ: consolidated criteria for reporting qualitative research
CP: consumer participant
GRIPP2: guidance for reporting involvement of patients and the public, version 2
N/A: not applicable
NSQHS: National safety and quality health service standards
PPI: patient and public involvement
RP: researcher participant
SM1: supporting materials 1
SP: staff participant

## 1 Introduction

Consumer and community involvement (CCI), also known as patient and public involvement (PPI), can enhance the quality and relevance of research, improve governance of healthcare services and support tailored, person‐centred healthcare [1, 2, 3, 4]. CCI involves partnering with past, current or future patients, or members of the public of a given community group, to collaboratively improve health and well‐being through research, or the evaluation, design or implementation of healthcare [5]. In Australia, partnering with consumers in the design and delivery of healthcare is one of the eight accreditation standards set by the National Safety and Quality Health Service Standards (NSQHS) [6], making it not just best practice but also standard practice. However, meaningful CCI partnerships can be undermined by poorly designed orientation processes and inaccessible systems that fail to meet the needs of consumers with diverse communication and support needs [7, 8, 9].

In healthcare partnering, orientation refers to the processes and resources used to introduce consumers to their role, organisational systems and available supports, and is important for enabling safe, informed and meaningful involvement. Mandatory training is an important component of orientation in health service settings because it supports patient and staff safety and the delivery of quality healthcare [10]. While consumers who partner with health services are not staff, orientation and elements of mandatory training are expected to ensure their safety and that of current patients, particularly where partnership activities take place within patient‐facing areas of the health service. For consumers who partner with health services, these orientation processes often include infection prevention and control training, orientation to remuneration processes, vaccination review, criminal history checks and general evacuation instructions [11]. However, infection control training for consumers is often adapted from resources developed for healthcare professionals and may not be appropriate for consumer audiences.

Consumers involved in partnering may have diverse communication, language and literacy needs, including people with low literacy or health literacy, people from culturally and linguistically diverse backgrounds and those with communication disorders. Aphasia, a language disorder commonly acquired after stroke, affects 30%–40% of acute stroke survivors [12] and provides one example of how conventional training approaches may create barriers to participation. When orientation and training rely on complex medical terminology and staff‐oriented systems, they may be inaccessible or intimidating for many consumers, reinforcing power imbalances, limiting relationship building and undermining partnerships [3, 13].

Co‐design is a participatory research approach that moves beyond consultation by involving consumers as partners in shaping the research process and contributing lived experience alongside other expertise [14]. In this study, a co‐designed approach was selected to enable consumers (lived‐experience experts), together with researchers and clinical staff (consumer partnering and infection control expertise), to collaboratively design orientation that is fit for purpose. Although several co‐designed training programmes have been developed for consumers, these have largely focused on supporting involvement once consumers are already engaged in partnership activities, such as involvement in research methods [14] or evaluating research materials [15], rather than on the orientation processes that enable participation in the first place [15, 16, 17]. As a result, a gap remains in the availability of accessible, mandatory training materials for consumers during orientation, particularly in infection prevention and control. Addressing this gap is important to ensure that orientation processes are fit for purpose for a diverse consumer population and do not unintentionally exclude those with different communication, language or literacy needs. This study aimed to explore experiences of orientation at a metropolitan rehabilitation hospital from the perspectives of consumers and staff, and to co‐design an accessible consumer‐focused infection control training module with consumers, staff and researchers from the same health service.

## 2 Methods

### 2.1 Study Design

This study used a qualitative methodology involving semi‐structured interviews and co‐design workshops. A constructivist‐interpretivist paradigm guided the analysis [18], acknowledging that researchers are part of the co‐creation of knowledge, and that their experiences, thoughts and ideas shape it. The Consolidated Criteria for Reporting Qualitative Research (COREQ) [19] and the Guidance for Reporting Involvement of Patients and the Public (GRIPP‐2) guidelines [20] informed reporting. Consumers were compensated for their time, and parking costs were covered.

### 2.2 Setting, Participants, Recruitment

Participants included consumers, staff and researchers from one research‐active metropolitan hospital in Queensland, the largest rehabilitation hospital in Australia. The hospital maintains an active CCI network, from which consumers were recruited. Eligible consumers had completed at least some orientation components. Consumers with diverse communication requirements, including people with communication difficulties, were purposively sampled to ensure that the design process considered a range of accessibility needs.

Eligible staff had expertise relevant to either infection control training, including members of the safety and quality team and specialist nurses, or to partnering with consumers at the hospital. Eligible researchers were those with consumer partnering experience at the hospital. Staff and researchers were recruited through professional CCI networks and the hospital Safety and Quality team. Purposive and convenience sampling were used to recruit individuals with relevant lived experience, as well as clinical, research and organisational expertise.

Staff and researchers were over‐recruited relative to consumers because they represented the several distinct expertise needed to inform the co‐design process and to account for staffing constraints, which were expected to affect their availability to attend workshop sessions. Consumer partners contributed lived experience of orientation and partnering with the health service. The co‐design process was structured to ensure that consumer perspectives directly informed design principles such as language, format and accessibility. Staff and research contributions were sought to inform clinical accuracy, feasibility, alignment with hospital infection‐control requirements and implementation.

### 2.3 Data Collection

#### 2.3.1 Semi‐Structured Interviews

Separate question guides were developed for staff and consumers, with interviews tailored to the area of expertise. The interview guides were informed by the study aims, existing hospital orientation and infection control training materials, the research team's experience in consumer partnering and infection control education, and usability and acceptability considerations. Interview guides are available in Supporting Materials SM1. The staff interview guide was piloted with one researcher, after which the question order and structure were modified to improve flow. Researchers participated only in workshops, not in interviews, due to their limited experience with consumer hospital orientation.

During interviews, participants viewed excerpts from current infection control training and commented on their suitability for consumers, including key content, design, accessibility and format. Interviews were completed online and audio‐recorded; transcripts were de‐identified and transcribed verbatim. The interviewer recorded brief field notes during and directly after interviews to document contextual observations and reflections relevant to the topic.

#### 2.3.2 Co‐Design Workshops

**Figure 1:**
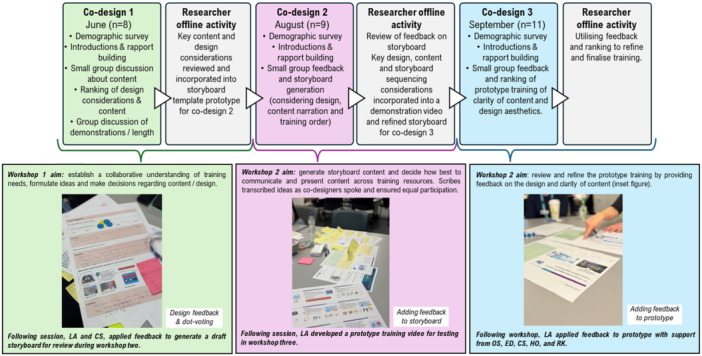
Co‐design process for infection control training development, including sample activities from co‐design workshops.

A series of three 2‐h co‐design workshops were conducted with relevant knowledge holders (Figure 1). Workshop co‐designers included consumers who had previously completed the interview component. Staff and researchers, some of whom had previously completed an interview. Co‐design methods were informed by the Metro North Co‐design Framework [21] and established co‐design principles. Consumer partnership guidance supported a clear purpose of involvement, attention to power and role expectations and reporting of consumers' contributions [22]. The workshops drew on participants' lived and professional experience to establish shared focus, collective critique and ideation in the production of knowledge [23].

Co‐design workshops were facilitated by LA, a researcher experienced in co‐design and supporting consumers, including those with communication difficulties. Structured facilitation techniques, including turn‐taking, open prompts, small‐group discussions and prioritisation activities, were supported by CS, HO, KB and ED, who also contributed as researcher co‐designers [21, 24]. This enabled equitable participation across consumers, staff and researchers and was consistent with health co‐design guidance emphasising structured involvement, shared decision‐making and equal partnership [25]. The facilitator and co‐facilitators met before and after workshops to debrief and reflect on small‐group dynamics, co‐design activities and facilitation. To support inclusive practices for co‐designer participants with aphasia, communication support strategies were implemented, such as aphasia‐friendly written and visual materials and additional time during activities [26]. Workshops began with a rapport‐building exercise and a summary of prior work to help balance power and develop shared understanding. No field notes were taken by facilitators during the workshops. Workshop data comprised co‐designer‐generated materials produced as outputs from the activities, such as written comments on printed frames, sticker dot voting, ranking outputs and storyboard annotations. Following each workshop, outputs were collated and applied to generate and refine resources for subsequent workshops (LA, CS, ED, HO). Following final refinements after workshop three, the content was reviewed by authors (RK, OS, JS) to confirm alignment with hospital infection control practices.

##### 2.3.2.1 Workshop One

Workshop one aimed to establish a collaborative understanding of training needs, to formulate ideas and decide on content and design. The workshop began with an introduction to infection control concepts and an overview of specific interview findings regarding the design of orientation and training. Interactive activities were used to elicit co‐designers' opinions and suggestions, including group discussions, sticker dot voting and writing individual and group suggestions on printed slides (Figure 1). Activities focused on discussing content and design considerations in small groups, followed by individual ranking of the importance of concepts for inclusion. Rankings were collated, shared and then discussed as a group to confirm content and design. A final activity considered the video's duration, content and format of any supporting materials. Following this session, LA and CS applied feedback to generate a draft storyboard for review during workshop two.

##### 2.3.2.2 Workshop Two

Workshop two aimed to generate storyboard content and decide how best to communicate and present content in the training. Opportunities were provided for individual and group comments regarding the suitability and ordering of content and design of each storyboard slide (Figure 1). Co‐designers received a draft storyboard scaffold with proposed content, along with several examples of images and icons to prompt discussion. Scribes captured ideas and ensured equal participation. The level of detail required to communicate key content and design considerations for each storyboard, including narration and images, was discussed in small groups. Further reflections focused on whether some concepts would be better suited to an accompanying handout. Ideas generated in the small group discussions were shared, and priority focus areas for workshop three were agreed by the whole group. Following this workshop, researchers (LA) developed a prototype training video.

##### 2.3.2.3 Workshop Three

Workshop three aimed to review and refine the prototype training video by providing feedback on its design and content clarity. Co‐designers viewed the prototype training and discussed their first impressions in small groups. They then individually ranked, using sticker dots on a 10‐point sliding scale, (1) clarity of content and (2) design aesthetics of each storyboard slide. Small groups subsequently provided feedback to improve elements of the training, with additional time dedicated to refining slides with lower clarity or aesthetics scores. The accuracy of the infection control principles and the minimum orientation requirements was confirmed by the authors (OS, JS) during the final workshop. Feedback from workshop three, including ranking outputs, group discussion notes and agreed refinements, was incorporated into the final version of the training resource.

### 2.4 Data Analysis

Initially, descriptive, inductive content analysis [27] was applied to questions related to the training design (LA, CS) from the semi‐structured interviews. This involved coding responses, grouping common concepts into categories and identifying themes related to design characteristics. This process was completed to inform the collation of a summary of themes presented to participants during co‐design workshop one. To more deeply analyse the experiences and insights of participants, transcripts were later thematically analysed following the steps outlined by Braun and Clarke [28]. The six steps of reflexive thematic analysis followed were familiarisation; coding; initial theme generation; theme review and development; theme refinement, naming and definition; and producing the report. Following familiarisation, researchers (ED, CS) independently coded a sample of staff and consumer interview transcripts. ED, CS, LA and HO reviewed and discussed the coded transcripts to consider alternative viewpoints before deciding on a consistent approach to coding of the entire dataset. Remaining transcripts were independently coded by CS, and queries were discussed with LA. Initial themes were generated by CS and LA. These were refined by clustering combinations of codes to understand patterns of meaning, followed by iterative rounds of generating and confirming code groupings and theme names against underlying quotes. Final themes and illustrative quotes were reviewed and refined by all authors. Coding was completed in NVivo 14.24.3, with theming managed in Microsoft Excel.

#### 2.4.1 Reflexivity

The interviewer (LA) was a qualified speech pathologist and experienced researcher in qualitative methodologies. ED, LA, KB, and HO are experienced qualitative researchers familiar with co‐design, participatory methods and reflexive thematic analysis. CS completed this work as part of her advanced undergraduate research thesis in speech pathology. All researchers worked at the hospital where the research was conducted, and their experiences were valued when considering alternative viewpoints when analysing the data. Some participants were known to the interviewer. To welcome open reflection and sharing of individual experience, participants were reassured of their anonymity and encouraged to express their opinions. Building rapport between researchers and participants can increase participants' willingness to share honestly [29]. Regular meetings and discussions throughout the thematic analysis process were used to promote critical reflection, challenge biases and enhance the reliability of findings.

## 3 Results

A total of nine participants (consumers *n* = 4; staff *n* = 5) attended an interview, and 14 (consumers *n* = 4; staff *n* = 6; researchers *n* = 4) contributed to co‐design. Not all participants contributed to all co‐design workshops. A summary of participant characteristics and contributions is provided in Table 1. One consumer participant had lived experience of mild expressive and receptive aphasia. Interviews were conducted online (May–June 2025). Staff interview duration: 34–45 min (mean = 39 min); consumer interviews: 44–64 min (mean = 51 min); co‐design workshops: 2 h each (June–September 2025).

**Table 1:** Participant demographics.

P#	Discipline	Years/months of experience in current role/consumer	Years/months of experience working with consumers total	Age	Gender	CALD† or communication differences	Interview	Co‐design‡
Staff/Researcher demographics								
SP1§	Allied Health	10 months	10 months	44	Female	Caucasian/English speaking	✔	✔ ✔
SP2	Administration Officer	7 months	2.5 years	43	Female	English as a second language	✔	
SP3	Allied Health	6 months	9 years	51	Female	Caucasian/English speaking	✔	✔ ✔
SP4	Clinical nurse	3.5 months	4.5 years	34	Female	Caucasian/English speaking	✔	✔
SP5	Clinical nurse	4.5 years	10 years	60	Female	Caucasian/English speaking	✔	✔ ✔
SP6	Clinical nurse	10 months			Male	Caucasian/English speaking		✔
RP1	Allied Health	20 years	20 years	49	Female	Caucasian/English speaking		✔ ✔ ✔
RP2	Allied Health	4 years	4 years	30	Female	Caucasian/English speaking		✔ ✔
RP3	Allied Health student	6 months	6 months	21	Female	Caucasian/English speaking		✔ ✔ ✔
RP4	Allied Health	2 years		38	Female	Immigrated from Sri Lanka/English speaking		✔
Consumer demographics								
CP1	Consumer partner	6–7 years	N/A	69	Male	N/A	✔	✔ ✔
CP2	Consumer partner	7 months	N/A	64	Female	N/A	✔	✔ ✔ ✔
CP3	Consumer partner	6–7 years	N/A	70–80	Female	N/A	✔	✔ ✔ ✔
CP4	Consumer partner	2 years	N/A	61	Male	Immigrated from New Zealand and Aphasia	✔	✔ ✔ ✔

*Note:* These demographics were all self‐reported by participants during interviews. If the participant did not attend an interview, these demographics were collected via survey at the start of the co‐design workshop.

^†^ Number of ✔ = number of co‐design workshops attended.

^‡^ CALD = culturally and linguistically diverse backgrounds.

^§^ SP = staff participant, RP = researcher participant, CP = consumer participant. Staff participant roles included project officer, patient experience officer, Safety & Quality Manager and researcher.

### 3.1 Qualitative Interview Themes

Two themes were generated from staff interviews regarding staff experiences participating in consumer infection control orientation. These were (1) Orientation lacks the flexible and tailored approach needed to engage consumers and (2) Rigidity of processes limits the diversity of consumers who complete orientation. From the consumer interviews, three themes were developed regarding their experiences with health service orientation. These were (1) orientation processes, training and materials are not accessible or tailored for consumer audiences; (2) orientation processes lack authentic, transparent partnership; and (3) mode of orientation processes and training can be restrictive or limit access.

#### 3.1.1 Staff Theme 1: Orientation Lacks the Flexible and Tailored Approach Needed to Engage Consumers

This theme captured the cumbersome, often inaccessible language associated with orientation paperwork, particularly for those with low literacy or limited access to technology. Face‐to‐face orientation was suggested to address this and facilitate rapport‐building, providing opportunities to ask questions and receive practical assistance, so consumers feel supported in completing the required onboarding paperwork. Some staff acknowledged that face‐to‐face orientation was not feasible for everyone and emphasised the importance of informing consumers about alternative ways to engage without on‐site attendance (e.g., online). Having the flexibility to align orientation processes with individual consumers' needs and preferences was considered a key component of person‐centred orientation. Additionally, having accessible consumer‐related information, such as a centralised consumer portal, was important for valuing CCI. Staff also emphasised the importance of valuing consumer contributions throughout the orientation process, suggesting that efficiency and keeping consumers informed supported this.

…I can go on as an employee of [hospital], there's a page, all the forms are there, it tells me what to do, who to send it to…if we really think about the orientation process from the consumer perspective, I think there's quite a bit of work to do…we'e probably come a long way in making this easier for staff to onboard consumers. (SP3) Staff emphasised the need for consumer training to differ from staff training; however, they expressed difficulty in determining consumers' understanding and how best to tailor information, ‘[it can be] …a fine line of educating and engaging them in infection control and not scaring them.’ (SP4). Concrete explanations and practical information, tailored to consumers, were identified as important for facilitating consumer understanding of complex infection control principles. However, opinions varied regarding what content should be included, such as whether consumers should be trained on ‘bare below the elbows’ or personal protective equipment.

#### 3.1.2 Staff Theme 2: Rigidity of Processes Limits the Diversity of Consumers Who Complete Orientation

This theme captured how rigid and restrictive orientation processes create barriers for consumers from culturally and linguistically diverse backgrounds, Aboriginal and/or Torres Strait Islander Peoples, those with physical disabilities, low literacy levels, difficulties accessing technology, those experiencing economic disadvantage or those living far from the hospital. Staff also reported that the rigidity and formality of orientation can be intimidating and confronting for consumers.

…I think the formality of the process scares a lot of those really vulnerable groups away, people who are more educated, generally white, articulate, those people aren't necessarily put off …when we add these layers of bureaucracy into the process, it adds more fear… I think it's because we do not make this process culturally suitable…

(SP3)

Vaccination compliance processes are ‘*probably the biggest one [barrier] I've come across in terms of getting people through the process*…’ (SP3), although staff also recognised that ‘*…that we do have a partnership role that we can play with people… you know, finding out if you're covered* [vaccination status] *or not…*’ (SP5). Staff identified that it is difficult for consumers to navigate costly, complex vaccination processes and that they require more support. However, they noted that service constraints, including staffing and costs, create barriers to providing support. Staff described the importance of providing transparent, accessible and respectful explanations about why certain information is being collected. Furthermore, staff identified that these processes need to become more flexible to accommodate diverse needs. ‘…*as a health service, we have to have systems and processes, but when those systems and processes get in the way of what our ultimate goal is, which is to engage these people*.’ (SP3)

#### 3.1.3 Consumer Theme 1: Orientation Processes, Training and Materials Are Not Accessible or Tailored for Consumer Audiences

Consumers identified that current orientation processes and infection control training are not tailored to their needs. They indicated that training should be practical, relevant and easily applied in context, highlighting the value of tangible examples that illustrate consequences:…*there's nothing more powerful than a message that shows when ‘you haven't done it right, this is what could happen …. because if we didn't have it or it wasn't done well, this is what could happen*.’ (CP2)

Additionally, consumers reported that excessive information and larger groups can be overwhelming, while smaller groups, hospital‐specific orientation, interactive discussions and opportunities to share experiences led to more personalised training. The concept of peer support and knowledge sharing was considered important, with hospital group orientation viewed as an opportunity to meet other consumers. Consumers highlighted the importance of staff acknowledging that infection control can be alarming, and cautioned that some consumers may have had negative hospital experiences, echoed by one consumer who recalled their experience of orientation ‘…*there were some opportunities at the meeting that were brought up…they were about infection control and I remember feeling quite scared and thinking “I cannot do this stuff ah I'll think about it” and I didn't volunteer*…’ (CP4)

Consumers described some training concepts as difficult for laypeople to understand, particularly for those from culturally and linguistically diverse backgrounds, as well as for those with communication difficulties or low literacy levels. ‘*Now aseptic, I remember googling aseptic again the other day, and I keep having to google aseptic…*’ (CP4). The importance of less jargon, simplified slides and more visuals was also emphasised.

#### 3.1.4 Consumer Theme 2: Orientation Processes Lack Authentic, Transparent Partnership

Consumers expressed a desire to be consulted, informed, partnered with and valued throughout orientation and in all aspects of CCI. Some consumers talked about their early experiences in the network, feeling unsure of their role as a consumer and appreciated staff support, ‘*…I didn't know really who what was, and I had no idea really what consumers actually did to be frank…’* (CP4). Consumers emphasised the importance of having their role explained to them in an accessible way. Staff support made consumers feel valued and was described as helpful for completing paperwork, identifying suitable projects, learning their CCI role and navigating complex vaccination compliance processes….*some of it [paperwork] was sent to me by email and then some were given to me at that meeting…and it was explained to me, some of it didn't need explanation, but some of it needed explanation, but I, particularly around the vaccination status…I felt like I had been informed at all times*. (CP2)

Consumers recognised a power differential between themselves and researchers; this was addressed by keeping them informed, empowering their participation and fostering a sense of belonging to the team. ‘*Yeah, you're staying informed and you feel like you're part of a team, you're not being used to do bits and pieces and then flicked off*…’ (CP1). This concept of empowerment through being informed was important throughout, from expression of interest, through orientation, to later partnership activities. Consumers also emphasised the importance of building transparent relationships and valuing contributions to avoid CCI becoming ‘…*tokenistic*…’ (CP3) or consumers feeling as though ‘…*I wanted to contribute, but I didn't feel that I was encouraged*.’ (CP3).

#### 3.1.5 Consumer Theme 3: Mode of Orientation Processes and Training Can be Restrictive or Limit Access

Consumers expressed differing opinions on the best mode of delivery for orientation and training. Face‐to‐face training was highlighted for building rapport with staff, enabling demonstrations of infection control practices and encouraging involvement, thereby supporting understanding. Most consumers considered face‐to‐face orientation and training to be preferable, but that ‘…*in‐person training is a luxury*.’ (CP3), acknowledging the importance of accessible online options when face‐to‐face is not practical. Hybrid arrangements, such as online training with optional face‐to‐face training, were proposed. Some consumers identified that online training may be more accessible, allowing them to re‐access information.

### 3.2 Co‐Design

Table 2 provides an overview of activities and refinements across the co‐design process and the co‐designer contributors.

**Table 2:** Finalised structure of co‐design workshops.

Workshop purpose	Activities	Aims and researcher offline activity	Participants†
1. Equalise power relationships, establish collaborative understanding of training needs and form decisions regarding content and design.	– Demographic survey. – Introductions and building rapport. *Training Content/Design Development* – Review content suggestions from interviews. – Small group discussions to decide if additional content should be included. – Voting on ranking scales to confirm importance of content suggestions from interviews. – Review design suggestions from interviews. – Small group discussions to decide what demonstrations should be included and what types/format of content should be included. – Voting on ranking scales to determine length of training video. – Whole group feedback and discussion.	*Aims* – List of key content to include in the training to be produced. – Design considerations to include. *Researcher offline activity* – Collation of information and feedback. – Development of storyboard prototype.	Researchers (*n* = 3), Staff (*n* = 2), Consumers (*n* = 3)
2. Validate training needs and resources and form decisions regarding how to communicate the content and considering the design of the training resources.	– Demographic survey. – Introductions and building rapport. – Overview of co‐design workshop one. *Storyboard Generation* – Considering the key concepts decided on during workshop one, drafting key content for each key message. – Deciding whether key concepts should be included in the training video or would be better suited to an accompanying handout. – Small group discussions on design considerations for each slide of the training (e.g., wording, images, video, narration). – Considering ordering of content within training. – Whole group discussion and feedback.	*Aims* – Ideas for how to communicate key concepts. – Considerations for the design of slides. *Researcher offline activity* – Collation of information and feedback. – Refine development of training resources.	Researchers (*n* = 2), Staff (*n* = 3), Consumers (*n* = 4)
3. Review prototype training and provide feedback on the design and clarity of content to refine final training.	– Demographic survey. – Introductions and building rapport. – Overview of prior work, feedback and discussions. *Prototype testing* – Watching prototype training twice as a whole group. – Small group discussions about initial impressions of training and broad feedback. – Voting on ranking scales of design and clarity of content of each training slide. – Small group discussions on design considerations and content for each slide of the training (e.g., wording, images, video, narration, ordering, how to refine content). – Whole group discussion and feedback. – Wrap‐Up and Next steps.	*Aims* – Feedback on the design and clarity of content of the prototype training. – Ideas for refinement of training *Researcher offline activity* – Collation of information and feedback. – Finalisation of training resources.	Researchers (*n* = 4), Staff (*n* = 3), Consumers (*n* = 4)

^†^ Two of the researchers in workshop one and three (RP2 and RP3) and one of the researchers in workshop two (RP3) also work clinically within the service.

#### 3.2.1 Content

Content aligned with the hospital's infection control, safety and quality accreditation standards, with concepts iteratively refined across workshops. Decisions about content focused on whether it helped consumers understand infection‐control principles and reduce risks. Two concepts considered necessary were how to wash hands effectively and when to stay home if unwell. During workshop two, content was consolidated and focused on nursing staff as the primary point of contact for consumers when entering patient spaces and interpreting hospital signage. A case example was suggested to illustrate mistakes and consequences, and how to mitigate these. In the final workshop, feedback rated the clarity of content as high (ranking = 8–9/10), with suggestions to expand the case study across more key messages to improve relevance and cohesion.

#### 3.2.2 Design

Design principles from the interviews were adapted across the co‐design process (Figure 1). Co‐designers offered varying suggestions on training length, from a series of ‘TikTok’ style videos to 15 min, with consensus reached on a 5‐min maximum. Image types (animations, icons, photos) were reviewed, with co‐designers considering real images more credible and easier to interpret than animated images/icons, although real images also posed a challenge for showing diversity. A decision was made that icons were too abstract, and a combination of animated and real images was ideal. The use of imagery that conveys conventional meaning was suggested, for example, green ticks and red crosses to indicate useful or harmful behaviours. During workshop three, after reviewing the prototype, co‐designers perceived that some graphics were too abstract and some real images too confronting. Agreement was reached on prioritising real images, adding slow narration and closed captions, reordering slides and bolding keywords to improve accessibility for communication.

Following this workshop, LA applied feedback to refine the training video with support (ED, HO, CS, OS, RK). The video (https://youtu.be/plPbfiPLJFc) educates consumers about infection control principles and responsibilities and supports decision‐making to keep themselves and others safe during hospital activities. Delivering the video during group orientation was recommended to provide opportunities for socialising and questions, and to develop an accompanying handout. Since its completion, the video has been implemented in the hospital with plans for wider use across the health service.

## 4 Discussion

This research explored the perspectives of consumers and staff about their experiences with consumer orientation and reports on the co‐design of an accessible infection control training resource for consumers. Rather than identifying orientation as a discrete administrative requirement, the findings suggest that orientation functions as an important entry point into consumer partnership. Consumers reported that orientation processes, including existing infection‐control training, were inflexible and not tailored to their needs, thereby limiting accessibility. They described a need for orientation and training that were clearer, more relevant and more responsive to different communication, language and literacy needs. Lack of adjustments, such as flexibility in communication and delivery modes, hindered genuine partnerships and risked excluding diverse consumers. Staff reinforced consumer perspectives and added that processes lack a person‐centred approach. This suggests that when orientation processes are inflexible, overly technical or poorly adapted to consumers' communication, language and literacy needs, they may undermine the principles of inclusion and partnership that consumer involvement seeks to promote. Taken together, the findings indicate that accessible orientation requires more than simplified information; it requires attention to the relational, practical and communication conditions that enable consumers to participate. These experiences informed the co‐design of a training video that is communication‐accessible, tailored to consumers and focused on the key principles of mandatory infection control training for consumers partnering with the hospital.

### 4.1 Challenges of Orientation

This study highlights the importance of tailoring orientation to consumers' needs. This aligns with two recent scoping reviews [2, 13]. Cox et al. explored consumer engagement in occupational therapy research, finding that consumer recruitment processes depended on consumers having high English literacy, internet access and higher socio‐economic backgrounds [2]. While this is consistent with our findings, our study identified additional barriers to consumer orientation relevant to the hospital environment, including vaccination procedures and diverse communication, language and literacy needs. These findings extend existing evidence by showing how institutional requirements designed to support safety and governance may unintentionally become barriers to participation when not adapted to consumers. In this way, orientation can reproduce exclusion even when the broader intent is to enable consumer involvement.

Proposed solutions to these barriers include hospital‐facilitated vaccination to reduce the burden on consumers and ensuring consumers who cannot come into the hospital are supported to engage in other ways. Ensuring accessible communication, especially for consumers from culturally and linguistically diverse backgrounds or those with communication needs, was strongly emphasised. These findings align with research indicating that inclusive and tailored communication with consumers can increase consumer diversity [13]. While studies highlight that complex jargon is intimidating to consumers and hampers interpersonal interactions [3, 13], there has been limited attention to co‐designing tailored consumer training on complex topics, such as infection control. This is an important gap because mandatory training topics are often treated as fixed content to be delivered rather than as topics that require adaptation to support meaningful engagement. In this study, staff and consumers reported the need to educate consumers on these crucial topics without frightening consumers, while still fostering appropriate caution. This highlights that training must communicate risk and responsibility, but if presented in a highly technical or fear‐based manner, it may discourage participation. A finding from staff perspectives was that they struggle to tailor training to consumer audiences, underscoring the importance of partnering with consumers to develop fit‐for‐purpose orientation and training.

### 4.2 Co‐Designing Accessible Training

Blair et al. [16] and Horobin et al. [15] co‐designed consumer training and highlighted the importance of adopting a person‐centred approach and incorporating social components. Our co‐design approach reflected this by applying a person‐centred approach to orientation and training, extending this with interactive mandatory training that promotes socialising with peers and opportunities to share experiences. The contribution of the present study lies in applying these principles to infection control orientation, a topic that is both mandatory and potentially complex for consumers. This demonstrates that even highly regulated or technical training content can be adapted through co‐design without losing its core safety purpose.

The co‐design process in our study enabled participants to expand on ideas from the interviews and engage in the iterative process of developing the training. As this study was conducted in a rehabilitation hospital, where some consumers may have communication disabilities, including aphasia, staff and consumers emphasised accessible communication that would support a broad range of consumers. Participants advocated for simple language, visual aids and brief, focused training, aligning with the principles of aphasia‐friendly resources [30, 31] and with broader principles of accessible health communication. The study also highlights the value of bringing staff and consumers together to develop orientation resources. Staff contributed knowledge of infection control requirements and organisational expectations, while consumers contributed experiential knowledge about what makes training understandable, acceptable and engaging. This combination helped ensure that the co‐designed final resource was fit for purpose from both institutional and consumer perspectives.

### 4.3 Significance

This research examined experiences associated with consumer orientation and co‐designed an accessible infection‐control training resource tailored for consumers in a metropolitan rehabilitation hospital setting. Implementing these findings could improve consumer experiences during orientation and support person‐centred consumer training. Reducing barriers to consumer orientation, such as simplifying jargon, using accessible design principles and offering flexible modes of engagement, may improve accessibility and promote involvement from a more diverse range of consumers, ensuring that health service initiatives better capture the needs of a broader cross‐section of the community.

The broader significance of these findings is that they reframe orientation as a part of the infrastructure of consumer involvement. Orientation is often positioned as an administrative process that occurs before consumers contribute. However, the design of orientation itself shapes who is able to participate, how prepared they feel and whether they experience the organisation as welcoming of their contribution.

### 4.4 Strengths and Limitations

A strength of this study was the attendance of consumer and researcher participants in co‐design workshops, along with the expertise of the contributors. Expertise included infection prevention and control, patient experience, research on communication impairments, consumer partnering and co‐production, consumer experience of hospital orientation and partnering within a rehabilitation research‐active hospital and lived experience of communication disability. This range of expertise strengthened the co‐process by enabling the resource to be informed by organisational, clinical, research and end‐user perspectives.

However, the composition of the co‐design group is also a limitation. Although consumers contributed across all workshops, they were fewer in number than staff/researchers and were older. This may have influenced workshop dynamics, including perceived authority and confidence to contribute. We sought to manage these dynamics through experienced, reflective facilitation, structured activities, small‐group discussions and accessible communication supports. Consumer feedback was prioritised in decisions about accessibility, format, language and delivery. However, future work should include a larger and more diverse consumer group.

This training was developed in a single hospital setting and may not translate directly to other settings with different infection control and orientation needs. Limitations include the small sample size, the presence of only one consumer with a communication disability and context‐specific orientation experiences. However, themes relating to the approach and attitudes towards orientation may be broadly applicable. In addition, the aphasia design principles relevant in this rehabilitation setting point to the value of accessible, flexible and consumer‐centred training and orientation for a wider range of consumers.

### 4.5 Future Directions

Future research should test and refine the co‐designed training with a broader sample of consumers, including those from culturally and linguistically diverse backgrounds, with low literacy, and with other support needs. This would extend consumer involvement literature that identifies these structural, communication and access barriers to participation [9, 13]. This research also found that orientation and mandatory training processes are important entry points for consumer involvement in health services. Future work could also build on these findings by examining whether the design principles identified in this study can be adapted to other healthcare settings, orientation requirements and mandatory training topics, contributing to stronger reporting and evaluation of how co‐design outputs are implemented in practice [4].

## 5 Conclusion

This research highlighted that many challenges exist in consumer orientation and training, including a lack of tailoring, person‐centredness, flexibility and authentic partnering. Training and orientation processes developed for staff may not be appropriate for consumers and may limit participation, particularly for people with diverse communication, language or literacy needs. Co‐designing orientation resources with consumers offers a practical way to develop more inclusive and responsive approaches. Strengthening consumer orientation to healthcare settings in this way may support more diverse consumer partnerships and improve partnering practices across healthcare settings.

## Author Contributions

**Clare Soeters:** writing – original draft, formal analysis, project administration, data curation. **Emmah Doig:** conceptualisation, supervision, writing – review and editing, methodology, data curation, formal analysis. **Hannah Olufson:** formal analysis, data curation, supervision, writing – review and editing, conceptualisation. **Kimberley A. Baxter:** writing – review and editing, validation, data curation. **Ruth King:** validation, writing – review and editing, data curation. **Olivia Sarri:** data curation, validation, writing – review and editing. **Joanne Sherring:** writing – review and editing, validation, data curation. **Lisa Anemaat:** conceptualisation, funding acquisition, writing – review and editing, formal analysis, project administration, supervision, data curation, resources, methodology.

## Funding

The authors have nothing to report.

## Ethics Statement

Ethical approvals were granted by Metro North (approval number: HREC/2025/MNH/115225) and the University of Queensland (approval number: 2025/HE000494) Human Research Ethics Committees. All participants provided written informed consent prior to participating.

## Conflicts of Interest

The authors declare no conflicts of interest.

## Supporting information


Supporting File 1

Supporting File 2

Supporting File 3

## Data Availability

The data that support the findings of this study are available on request from the corresponding author. The data are not publicly available due to privacy or ethical restrictions. https://authorservices.wiley.com/author‐resources/Journal‐Authors/open‐access/data‐sharing‐citation/data‐sharing‐policy.html.
