# Supplementary material for: Improving the Accessibility of Consumer Orientation: Co‐Designing Infection Control Training for Consumers Partnering With Health Services
Source: Health Expect. 2026 Jul 28;29(4):e70792. doi: 10.1111/hex.70792 (PMC13411796; doi:10.1111/hex.70792)
Supplement: Supplementary file 2 — Supporting File 2 [file HEX-29-e70792-s003.pdf]

# GRIPP2 short form

| Section and topic | Item | Reported on page No |
|---|---|---|
| 1: Aim | Report the aim of PPI in the study | 2 |
| 2: Methods | Provide a clear description of the methods used for PPI in the study | 2-4 |
| 3: Study results | Outcomes—Report the results of PPI in the study, including both positive and negative outcomes | n/a |
| 4: Discussion and conclusions | Outcomes—Comment on the extent to which PPI influenced the study overall. Describe positive and negative effects | 5-8, 9, 10-11 |
| 5: Reflections/critical perspective | Comment critically on the study, reflecting on the things that went well and those that did not, so others can learn from this experience | 11 |

Staniszewska S, Brett J, Simera I, et al. GRIPP2 reporting checklists: tools to improve reporting of patient and public involvement in research. *BMJ.* 2017;358:j3453.
