# Supplementary material for: Improving the Accessibility of Consumer Orientation: Co‐Designing Infection Control Training for Consumers Partnering With Health Services
Source: Health Expect. 2026 Jul 28;29(4):e70792. doi: 10.1111/hex.70792 (PMC13411796; doi:10.1111/hex.70792)
Supplement: Supplementary file 3 — Supporting File 3 [file HEX-29-e70792-s002.pdf]

SM1. Interview guides

Consumer Interview Guide

Section	Questions
Experiences with onboarding processes	1. Can you tell me a little about your experience joining the STARS consumer network?<br>*Prompts:*<br>• What do you think worked well?<br>• What might be improved?<br>2. Did you attend an orientation day at STARS as part of your onboarding?<br>*Prompts:*<br>• Can you tell me a little about this experience?<br>• Did you complete any training as part of this process?<br>3. Did you complete any training (mandatory training) as a part of this process?<br>*Prompts:*<br>• Can you tell me a little about this experience?<br>• What do you think worked well?<br>• What might be improved?
Feedback and opinions on current consumer infection control training material (participant shown 4 slides from the current training slide deck)	1. After looking at these images, do you have any thoughts on the:<br>• Content?<br>• Design?<br>• How do you think this training should be delivered (e.g., in person, online, other)?<br>*Additional Prompts:*<br>2. If we were going to design this information for new consumers, based on your experience, what do you think is important for consumers to know?<br>3. Are there any other thoughts that you have on how this information should be structured?
Open discussion	1. Is there anything else that you would like to add?

Staff Interview Guide (1) Infection control

**Section**	**Questions**
Experiences with onboarding processes	1. Can you tell me a little about the mandatory training you have delivered as part of your role?<br>*Prompts:*<br>• What do you think worked well?<br>• What might be improved?<br>2. Have you delivered any mandatory training to consumers? – describe?<br>*Prompts:*<br>• What do you think worked well / barriers (for you / consumers)?<br>• Have consumers been involved in the process?<br>• Design considerations for consumers?<br>3. What are the key components of the infection control mandatory training which need to be addressed to meet NSQHS standards for consumers?<br>*Prompts:*<br>• What do you think needs to be improved in the way we deliver mandatory training to consumer partners?<br>4. Were there any strategies you used (or think you should use) to adapt the delivery of the training for consumer partners with communication difficulties in particular?
Feedback and opinions on current consumer infection control training (participant shown 4 slides from the current training slide deck)	1. Here is an example of some of the slides from the current infection control mandatory training materials. Taking a look at these images, do you have any thoughts on the content / design / accessibility of these materials for a consumer/lay audience?<br>2. Are there any other thoughts that you have on how this information should be structured?
Open discussion	1. Is there anything else that you would like to add?

Staff Interview Guide (2) Patient Experience

<table>
<tr><th>Section</th><th>Questions</th></tr>
<tr><td>Experiences with onboarding processes</td><td>1. Can you tell me a little about your experience with consumers?
Prompts:
• What do you think worked well?
• What might be improved?
2. Have you been involved in an orientation day at STARS as part of consumer onboarding?
Prompts:
• Can you tell me a little about this experience?
• Did you complete any training as part of this process?
3. Have you delivered any mandatory training to consumers? – describe?
Prompts:
• What do you think worked well / barriers (for you / consumers)?
• Have consumers been involved in the process?
• Design considerations for consumers?
4. What do you think needs to be improved in the way we deliver mandatory training to consumer partners?
5. Were there any strategies you used (or think should be used) to adapt the delivery of the training for consumer partners with communication difficulties in particular?</td></tr>
<tr><td>Feedback and opinions on current consumer infection control training (participant shown 4 slides from the current training slide deck)</td><td>1. Here is an example of some of the slides from the current infection control mandatory training materials. Taking a look at these images, do you have any thoughts on the content / design / accessibility of these materials for a consumer/lay audience?
2. Are there any other thoughts that you have on how this information should be structured?</td></tr>
<tr><td>Open discussion</td><td>1. Is there anything else that you would like to add?</td></tr>
</table>
